# The complete mitochondrial genome data of *Cavenderia pseudoaureostipes*

**DOI:** 10.1016/j.dib.2026.113236

**Published:** 2026-09-04

**Authors:** Kamonchat Prommarit, Thanyaporn Chittavichai, Supanut Utthiya, Supachai Vuttipongchaikij, Passorn Wonnapinij

**Affiliations:** a Department of Genetics, Faculty of Science, Kasetsart University, Bangkok 10900, Thailand; b Omics Center for Agriculture, Bioresources, Food and Health, Kasetsart University (OmiKU), Bangkok 10900, Thailand

**Keywords:** Social amoeba, Mitogenome, Sequencing, Thailand

## Abstract

*Cavenderia pseudoaureostipes*, a dictyostelid species from Thailand described in 2018, is distinguished by the size and complexity of its fruiting bodies. Here, we report its complete mitochondrial genome, assembled from Illumina short-read data. The AT-rich circular genome is 68,470 bp in length, making it larger than previously reported dictyostelid mitogenomes. It contains 41 protein-coding genes, 17 tRNA genes, and three rRNA genes, all encoded on a single strand. Introns were detected in *cox1/2* and *cytb*, and the genome exhibits the conserved A–B–C segmental arrangement characteristic of *Cavenderia*. Phylogenetic analysis based on 30 conserved protein-coding genes supported the monophyly of *Cavenderia* and other genera, consistent with order-level classifications. Within *Cavenderia, C. pseudoaureostipes* was most closely related to *C. subdiscoidea*, in agreement with the SSU-based phylogeny. This mitogenome represents a valuable resource for comparative which will help resolving key questions regarding evolution of dictyostelids mitochondrial genome.

Specifications Table

**Table:** 

Subject	Biology
Specific subject area	Molecular systematics
Type of data	Raw, Analyzed
Data collection	*Cavenderia pseudoaureostipes* was obtained from the Dicty Stock Center (accession no DBS0350781). Genomic DNA was extracted from aggregation-stage cells and sequenced using paired-end 150 bp reads on an Illumina platform. Raw sequencing reads were deposited in the Sequence Read Archive (SRA) under BioProject PRJNA1417712. The mitochondrial genome was assembled with Unicycler and annotated using BLASTx/BLASTn and tRNAscan-SE.
Data source location	*Cavenderia pseudoaureostipes* was first collected by Steven L. Stephenson at Wat Pong AO Temple, Mae Chan District, Chiang Rai Province, Thailand [1]. John C. Landolt later deposited the isolate in the Dicty Stock Center, Northwestern University, Chicago, IL, USA (http://dictybase.org/StockCenter/StockCenter.html).
Data accessibility	Repository name: NCBI Genbank Data identification number: OP749982.1 Direct URL to data: https://www.ncbi.nlm.nih.gov/nuccore/OP749982.1
Related research article	None

## 1 Value of the Data

• This study reports, for the first time, the complete mitochondrial genome sequence of *Cavenderia pseudoaureostipes*, a recently described species among the nine *Cavenderia* species identified in Thailand [1,2] and 30 species recognized worldwide [2]. *C. pseudoaureostipes* is characterized by relatively large and complex fruiting bodies compared with other members of the genus [1].
• Mitochondrial genome sequences are commonly used in evolutionary and comparative genomic studies. In Dictyostelia, mitochondrial DNA analyses contribute to the characterization of gene content, genome organization, and mitochondrial roles during development [3,4].
• Key questions remain unresolved, including the mechanisms underlying segmental rearrangements and the ancestral state of codon usage. Additional complete mitochondrial genome sequences are needed to support comparative analyses of segmental rearrangements and codon usage patterns in Dictyostelia. The complete mitogenome sequence of *C. pseudoaureostipes* provides sequence data that can be used in future studies of mitochondrial genome evolution.

## 2 Background

Dictyostelia (cellular slime molds) are bacterivorous amoebozoans widely distributed in soil and leaf litter, where they play important ecological roles [1,2]. Their life cycle alternates between unicellular and multicellular stages, with the multicellular sorocarpic cycle being the most intensively studied and taxonomically informative [5]. Fruiting body morphology, representing the most complex developmental stage, remains a central criterion for Dictyostelia classification.

*Cavenderia pseudoaureostipes* is one of five dictyostelid species newly described from northern Thailand [1]. It exhibits distinctive violaceum-type aggregation, migrating pseudoplasmodia, and variable fruiting body morphologies, including clustered, prostrate, and solitary unbranched sorocarps. Although its 18S rDNA sequence is available in GenBank, historical misannotation reflects outdated taxonomic classification at the time of sequence submission, before the recognition of twelve genera and formal species descriptions [1,6].

Complete mitochondrial genomes provide additional sequence information that can complement 18S rDNA data in species-level taxonomic studies [7] and for investigating mitochondrial genome evolution. In Dictyostelia, mitogenome sequences can be used in comparative analyses of gene content, genome organization, codon usage, and genome rearrangements [4,8]. The complete mitogenome sequence of *C. pseudoaureostipes* adds to the available mitochondrial genome data for Dictyostelia and may facilitate future comparative and taxonomic studies.

## 3 Data description

### 3.1 Characteristics of *Cavenderia pseudoaureostipes’* mitochondrial genome

**Fig. 1:**
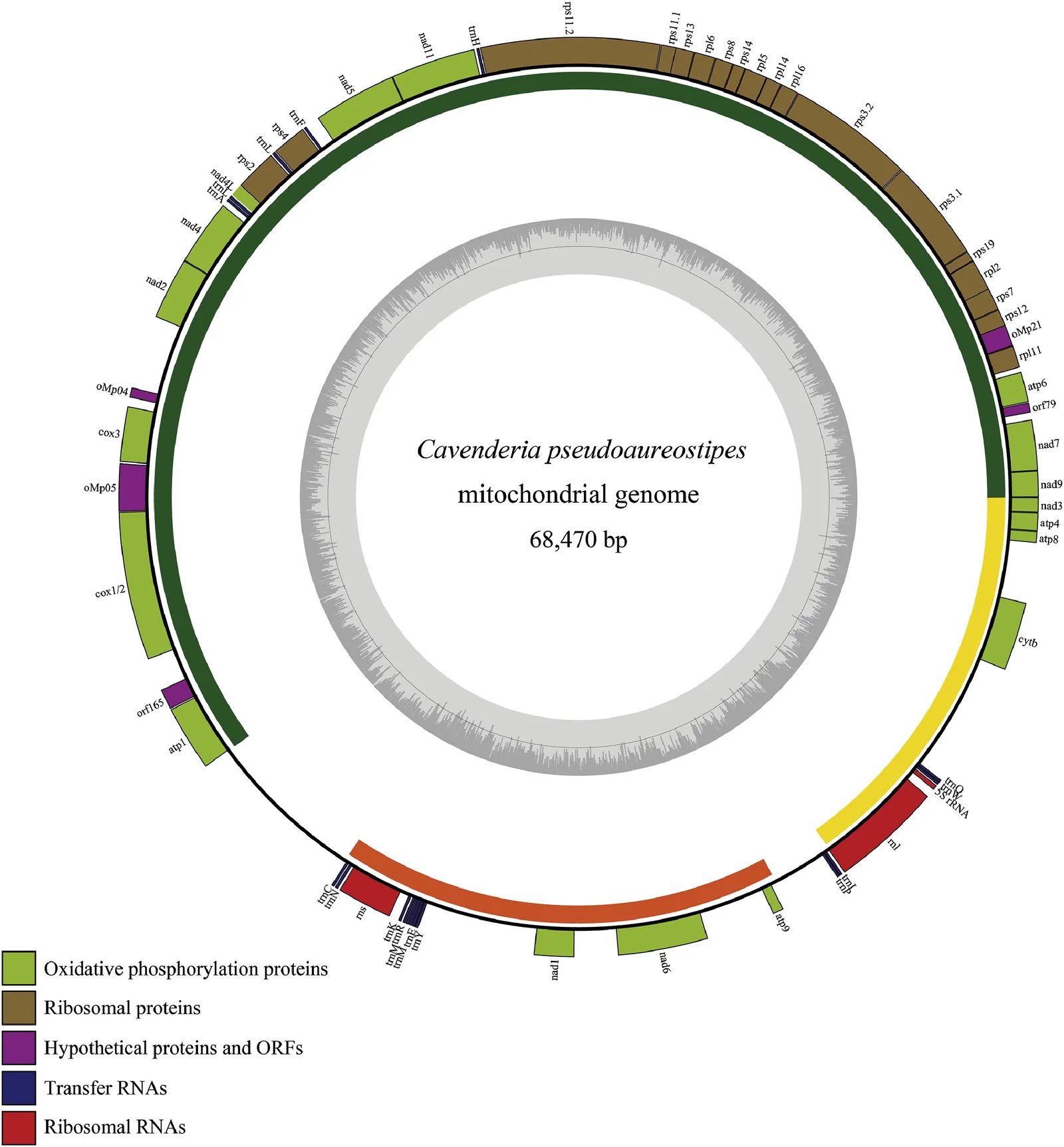
Gene annotation map *C. pseudoaureostipes* mitochondrial genome (GenBank accession number OP749982.1). Genes are shown on a circular map. Protein-coding genes are color-coded by functional category: oxidative phosphorylation (green), ribosomal proteins (dark brown), and hypothetical proteins and ORFs (purple). Transfer RNA (blue) and ribosomal RNA genes (red) are indicated separately. The grey inner circle represents GC content variation across the genome. The genome is structured into three conserved segments: A (*nad9–atp1*), B (*trnC–atp9*), and C (*rnl–nad3).* In this species, the segmental arrangement follows the A–B–C pattern, represented by the thick lines in dark green (A), orange (B), and yellow (C), respectively.

The complete mitochondrial genome of *C. pseudoaureostipes* was assembled from whole-genome Illumina sequencing data, yielding a circular genome of 68,470 bp. The nucleotide composition consists of 44.33% A, 31.70% T, 8.43% C, and 15.54% G. All genes are encoded on a single strand and include three ribosomal RNA genes — small subunit rRNA (*rns*), large subunit rRNA (*rnl*), and *5S rRNA —* 17 transfer RNA genes, and 41 protein-coding genes. Among the protein-coding genes, 18 are associated with oxidative phosphorylation, 18 encode ribosomal proteins, and 5 encode hypothetical proteins (Table 1, Fig. 1). The *cox1/2* and *cytb* genes each contain a single intron. As in other dictyostelid mitogenomes, the genes *rps3.2* and *rps11.2* were identified.

**Table 1:** Mitochondrial genome features of *C. pseudoaureostipes* (GenBank Accession number: OP749982.1).

Gene	Product	Start	End	Orientation	Length (base pair)
*nad9*	NADH dehydrogenase subunit 9	1	624	forward	624
*nad7*	NADH dehydrogenase subunit 7	628	1848	forward	1221
*orf79*	hypothetical protein	2019	2258	forward	240
*atp6*	ATPase subunit 6	2273	3007	forward	735
*rpl11*	ribosomal protein L11	3121	3654	forward	534
*oMp21*	mp21-like protein	3659	4195	forward	537
*rps12*	ribosomal protein S12	4167	4577	forward	411
*rps7*	ribosomal protein S7	4584	5129	forward	546
*rpl2*	ribosomal protein L2	5105	5884	forward	780
*rps19*	ribosomal protein S19	5887	6141	forward	255
*rps3.1*	ribosomal protein S3	6148	8628	forward	2481
*rps3.2*	ribosomal protein S3	8640	11,687	forward	3048
*rpl16*	ribosomal protein L16	11,696	12,136	forward	441
*rpl14*	ribosomal protein L14	12,142	12,519	forward	378
*rpl5*	ribosomal protein L5	12,521	13,069	forward	549
*rps14*	ribosomal protein S14	13,074	13,376	forward	303
*rps8*	ribosomal protein S8	13,382	13,813	forward	432
*rpl6*	ribosomal protein L6	13,822	14,337	forward	516
*rps13*	ribosomal protein S13	14,343	14,843	forward	501
*rps11.1*	ribosomal protein S11	14,783	15,145	forward	363
*rps11.2*	ribosomal protein S11	15,158	19,468	forward	4311
*trnH*	tRNA-His	19,490	19,561	forward	72
*nad11*	NADH dehydrogenase subunit 11	19,618	21,666	forward	2049
*nad5*	NADH dehydrogenase subunit 5	21,672	23,687	forward	2016
*trnF*	tRNA-Phe	24,028	24,098	forward	71
*rps4*	ribosomal protein S4	24,129	24,962	forward	834
*trnL*	tRNA-Leu	24,981	25,063	forward	83
*rps2*	ribosomal protein S2	25,100	26,116	forward	1017
*nad4L*	NADH dehydrogenase subunit 4L	26,120	26,413	forward	294
*trnL*	tRNA-Leu	26,446	26,527	forward	82
*trnA*	tRNA-Ala	26,540	26,611	forward	72
*nad4*	NADH dehydrogenase subunit 4	26,743	28,344	forward	1602
*nad2*	NADH dehydrogenase subunit 2	28,351	29,841	forward	1491
*oMp04*	mp04-like protein	31,612	31,851	forward	240
*cox3*	cytochrome oxidase subunit 3	32,071	33,396	forward	1326
*oMp05*	mp05-like protein	33,431	34,579	forward	1149
*cox1/2*^a^	cytochrome oxidase subunit 1 and subunit 2	34,591	38,162	forward	3572
*orf165*	hypothetical protein	38,951	39,448	forward	498
*atp1*	ATPase subunit 1	39,470	41,053	forward	1584
*trnC*	tRNA-Cys	45,171	45,241	forward	71
*trnN*	tRNA-Asn	45,265	45,343	forward	79
*rns*	small subunit ribosomal RNA	45,397	46,731	forward	1335
*trnK*	tRNA-Lys	46,959	47,031	forward	73
*trnR*	tRNA-Arg	47,070	47,142	forward	73
*trnM*	tRNA-Met	47,150	47,222	forward	73
*trnM*	tRNA-Met	47,229	47,299	forward	71
*trnE*	tRNA-Glu	47,305	47,378	forward	74
*trnY*	tRNA-Tyr	47,382	47,465	forward	84
*nad1*	NADH dehydrogenase subunit 1	50,290	51,249	forward	960
*nad6*	NADH dehydrogenase subunit 6	52,294	54,456	forward	2163
*atp9*	ATPase subunit 9	56,114	56,377	forward	264
*trnP*	tRNA-Pro	57,864	57,935	forward	72
*trnI*	tRNA-Ile	57,937	58,010	forward	74
*rnl*^b^	large subunit ribosomal RNA	58,069	60,844	forward	2776
*5S rRNA*	5S ribosomal RNA	60,955	61,076	forward	122
*trnW*	tRNA-Trp	61,104	61,175	forward	72
*trnQ*	tRNA-Gln	61,179	61,250	forward	72
*cytb*^a^	cytochrome b	64,280	65,937	forward	1658
*atp8*	ATPase subunit 8	67,382	67,657	forward	276
*atp4*	ATPase subunit 4	67,665	68,099	forward	435
*nad3*	NADH dehydrogenase subunit 3	68,105	68,467	forward	363

a *cox1/2* and *cytb* contain introns.

b The range of *rnl* differs slightly from that shown in the GenBank database.

Dictyostelid mitogenomes were first noted to exhibit segmental rearrangement by Heidel and Glöckner (2008), who identified two segments whose relative order differs between genera [8]. This analysis was subsequently refined to three conserved segments — A (*nad9–atp1*), B (*trnC–atp9*), and C (*rnl–nad3*) — with two arrangement patterns: the ancestral A–B–C pattern, found in *Cavenderia, Heterostelium*, and other non-*Dictyostelium* genera, and the derived A–C–B pattern, restricted to *Dictyostelium* species (except *D. purpureum*, which retains the ancestral pattern) [4]. Phylogenetic analysis indicates that the transposition of segments B and C occurred after *D. purpureum* diverged from other *Dictyostelium* lineages [4]. Heidel and Glöckner (2008) proposed that recombination drives this segmental transposition, with tRNA genes potentially mediating the rearrangement; this hypothesis is supported by the observation that most tRNA gene displacements in dictyostelid mitogenomes occur in segment B, particularly at its 3′ end [4]. The mitogenome of *C. pseudoaureostipes* exhibits the ancestral A–B–C arrangement consistent with other *Cavenderia* species, including *C. fasciculata* (NC_010653) and *C. subdiscoidea* (OP168900).

Comparisons with other *Cavenderia* mitogenomes showed differences in genome length and intergenic region size. For example, while the intron distribution in *C. pseudoaureostipes* (*cox1/2* and *cytb*) parallels that of *C. fasciculata*, the total genome length is slightly larger. *rps3.2* and *rps11.2* were present in *Cavenderia* mitogenomes but were not detected in some *Dictyostelium* species [4]. These comparisons highlight that, *C. pseudoaureostipes* shares a conserved mitogenome architecture with other *Cavenderia* species but differs in genome length and intron distribution.

### 3.2 Phylogenetic analysis

**Fig. 2:**
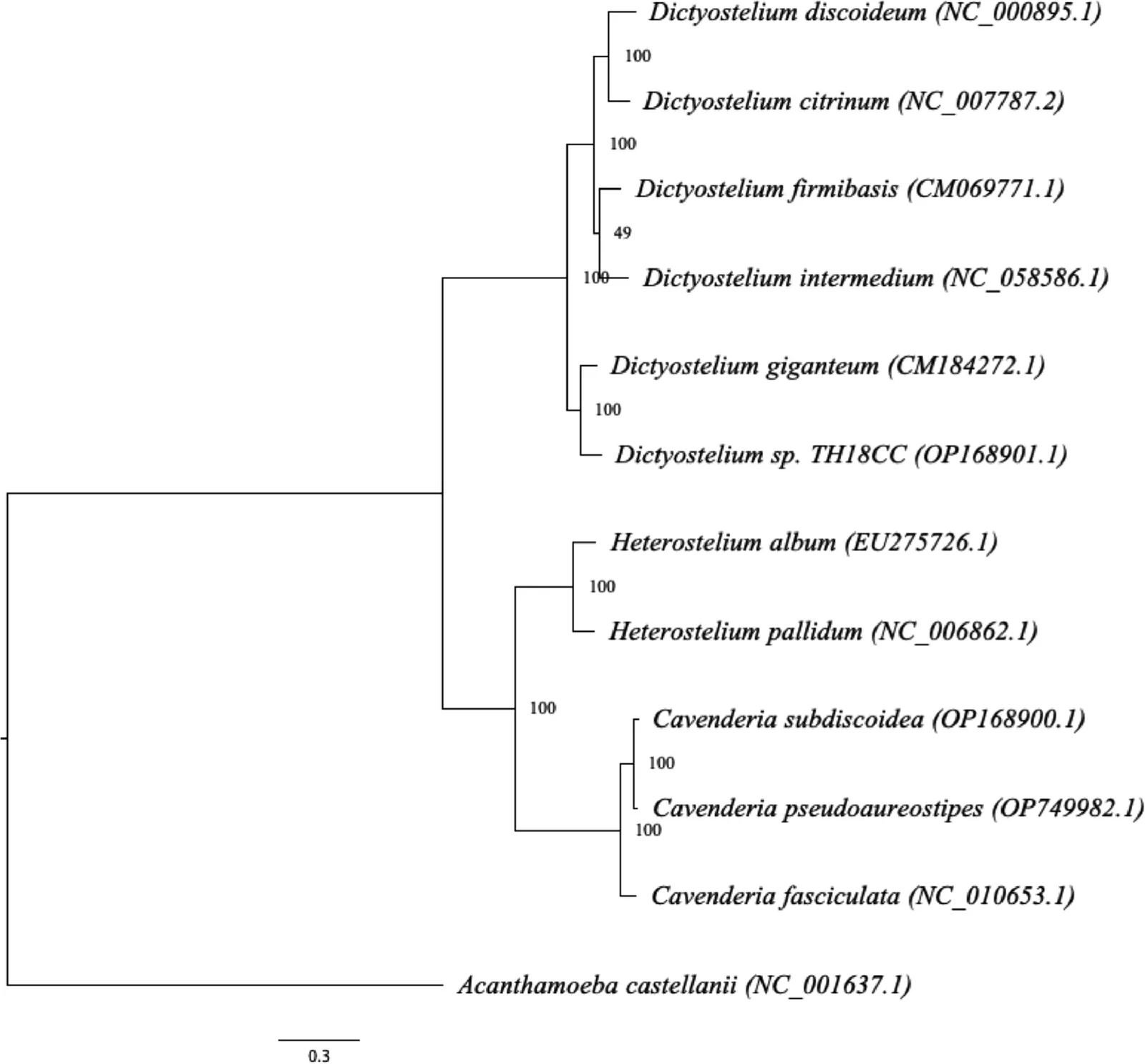
Phylogenetic tree based on 30 mitochondrial protein-coding genes using the maximum likelihood (ML) method with the LG+G+F substitution model and 1000 bootstrap replicates.

Phylogenetic analysis based on 30 mitochondrial protein-coding genes from 12 species, using *Acanthamoeba castellanii* as the outgroup, separated *Dictyostelium, Heterostelium*, and *Cavenderia* into two major groups [6]. For *D. firmibasis* (CM069771.1) and *D. giganteum* (CM184272.1), whose GenBank records contain incomplete mitochondrial annotations, the 30 protein-coding genes used in this analysis were re-identified; all 30 genes were among the 36 and 38 mitochondrial protein-coding genes reported for *D. giganteum* and *D. firmibasis*, respectively [9]. Within *Dictyostelium*, two monophyletic clades were recovered: one comprising *D. giganteum* and *Dictyostelium* sp. TH18CC*,* and the other comprising the remaining *Dictyostelium* species. *D. firmibasis* was closely related to *D. intermedium. C. pseudoaureostipes* clustered with *Cavenderia* species with high bootstrap support (Fig. 2). This clade is grouped with the monophyletic clade of *Heterostelium*, together comprising the order Acytosteliales*.* Within *Cavenderia, C. pseudoaureostipes* was more closely related to *C. subdiscoidea* than to *C. fasciculata.* The relationship among *Cavenderia* species was consistent with SSU-based phylogeny [1]. At the order level, this topology is concordant with nuclear phylogenies based on 18S rDNA [6] and multi-gene nuclear protein-coding sequences [10], which similarly support the separation of Dictyosteliales and Acytosteliales and the monophyly of *Cavenderia.* The mitogenome of *C. pseudoaureostipes* can be used in future comparative and phylogenetic analyses within Dictyostelia.

## 4 Experimental Design, Materials and Methods

### 4.1 Sample collection

*Cavenderia pseudoaureostipes* used in this study was originally collected in Chiang Rai Province, Thailand, and described morphologically by Vadell et al. (2018). It was deposited in the Dicty Stock Center by John C. Landolt and obtained for this work under accession number DBS0350781.

### 4.2 DNA extraction and sequencing

Genomic DNA (gDNA) was isolated from *C. pseudoaureostipes* cells at the aggregation stage following the protocol described by Utthiya et al. (2022) [11]. The quality and concentration of the extracted gDNA were assessed using agarose gel electrophoresis and a NanoDrop spectrophotometer (Thermo Scientific). High-quality gDNA was then submitted to a sequencing service for library preparation and sequencing on the Illumina platform. Sequencing was performed using a paired-end 150 bp strategy, generating approximately 12 Gb of paired-end reads for mitochondrial genome assembly.

### 4.3 Mitochondrial genome assembly and annotation

The quality of short-read data was assessed using FastQC [12]. Because dictyostelid amoebae are cultured on bacteria as a food source, bacterial DNA from the food organism contaminates total DNA preparations. To remove this contamination*,* reads were mapped to the *Escherichia coli* reference genome (NC_000913), the bacterium used for culturing *C. pseudoaureostipes,* with BWA-MEM [13]. Reads that mapped to the *E. coli* genome were discarded, and only unmapped reads were retained for mitochondrial genome assembly with Unicycler [14]. The resulting circularized mitogenome was visualized with OGDRAW [15].

Protein-coding genes were annotated using BLASTx [16] against a local database containing annotated proteins from five dictyostelid species: *Dictyostelium discoideum* (NC_000895)*, D. citrinum* (NC_007787.2)*, Heterostelium pallidum* (NC_006862)*, H. album* (EU275726)*,* and *Cavenderia fasciculata* (NC_010653). Exon–intron structures of *cox1/2, atp9*, and *cytb* were manually characterized using multiple sequence alignment with MUSCLE [17]. Intergenic regions lacking annotation were further examined using ORFfinder (https://www.ncbi.nlm.nih.gov/orffinder/) and BLASTp searches against the non-redundant protein database. ORFs showing >50% query coverage and ≥30% identity to Dictyostelia hypothetical proteins were annotated as hypothetical proteins.

To include *D. firmibasis* and *D. giganteum* in the mitochondrial phylogenetic analysis, their mitochondrial genome sequences were retrieved from GenBank (CM069771.1 and CM184272.1, respectively). Both records originate from whole-genome shotgun assemblies and contain incomplete mitochondrial protein-coding gene annotations. Protein-coding genes were therefore re-identified using BLASTx searches against the local database of five dictyostelid mitochondrial genomes described above.

Transfer RNA genes were predicted using tRNAscan-SE [18]. Ribosomal RNA genes, including the small subunit rRNA (*rns*), large subunit rRNA (*rnl*), and 5S rRNA, were identified using BLASTn [16] against a local database containing ribosomal RNA sequences from the same five dictyostelid species. Exon–intron structure of the *rnl* gene was manually examined and characterized through multiple sequence alignment with MUSCLE [17].

### 4.4 Phylogenetic analysis

The complete mitochondrial genome of *C. pseudoaureostipes* was analyzed in conjunction with ten other dictyostelid species, with *Acanthamoeba castellanii* serving as the outgroup. Amino acid sequences of 30 single-copy orthologous protein-coding genes (*nad9, nad7, atp6, rpl11, rps12, rps7, rpl2, rps19, rpl16, rpl14, rpl5, rps14, rps8, rpl6, rps13, nad11, nad5, rps4, rps2, nad4L, nad4, nad2, cox3, cox1/2, atp1, nad1, nad6, atp9, cytb,* and *nad3*) were aligned using MUSCLE [17]. Alignments were inspected and manually adjusted in AliView [19] before concatenation with catfasta2phyml (https://github.com/nylander/catfasta2phyml) and used as input for phylogenetic analysis.

The best-fit substitution model was selected using ModelTest-NG (https://github.com/ddarriba/modeltest) under the AICc criterion, which identified LG+G+F. A maximum likelihood phylogenetic tree was constructed with RAxML [20] using this model and 1000 bootstrap replicates.

## Limitations

None.

## Ethics Statement

Authors have read and follow the ethical requirements for publication in Data in Brief and confirming that the current work does not involve human subjects, animal experiments, or any data collected from social media platforms.

## CRediT Author Statement

**Kamonchat Prommarit**: Methodology, Formal analysis, Data curation, Visualization, Writing - Original Draft. **Thanyaporn Chittavichai**: Investigation, Visualization. **Supanut Utthiya**: Investigation. **Supachai Vuttipongchaikij**: Resources. **Passorn Wonnapinij**: Conceptualization, Methodology, Writing - Review & Editing, Supervision, Funding acquisition.

## Declaration of Generative AI and AI-Assisted Technologies in the Manuscript Preparation Process

During the preparation of this work the authors used Biomni (Phylo) to assist with interpreting reviewer comments and drafting revisions, and ChatGPT (GPT-5.2) to improve the clarity and readability of the manuscript. After using these tools, the authors reviewed and edited the content as needed and take full responsibility for the content of the published article.

## Data Availability

Mendeley DataThe complete mitochondrial genome of Cavenderia pseudoaureostipes (Original data)
